# Editorial: Pain-related neural networks and regulation mechanisms

**DOI:** 10.3389/fnmol.2022.1051335

**Published:** 2022-10-07

**Authors:** Wen Wu, Jie Jia, Fengxian Li, Weiwei Peng, Howe Liu, Hui Chen, Yu Shi

**Affiliations:** ^1^Department of Rehabilitation, Zhujiang Hospital, Southern Medical University, Guangzhou, China; ^2^Department of Rehabilitation Medicine, Huashan Hospital, Fudan University, Shanghai, China; ^3^Department of Anesthesiology, Zhujiang Hospital, Southern Medical University, Guangzhou, China; ^4^School of Psychology, Shenzhen University, Shenzhen, China; ^5^Department of Physical Therapy, Allen College, Waterloo, IA, United States

**Keywords:** pain, neural mechanisms, neural regulation, multiple omics, biomarker, machine learning

Pain is defined as an unpleasant sensory and emotional experience associated with, or resembling actual or potential tissue damage. However, the pain-related neural networks and regulation mechanisms are still not clearly understood, particularly in the thematic areas of pain biomarkers in multi-omics, molecular mechanism of pain comorbidity, exploration of pain sensitivity, application of machine learning (ML) in solving specific pain problems, and the prospect of research trends in the field of pain. So, we conducted such topical research with a total of 14 articles, including one review, one study protocol, two bibliometric analysis studies, and 10 original quantitative studies.

## Biomarkers in pain pathogenesis

The use of high-throughput screening and epigenetic study provides evidence to elucidate the pathogenesis and pathophysiology of disease. Screening biomarkers through multi-omics data is an important approach to study pain-related neural mechanisms. Song et al. explored the effect of swimming exercise on the chronic constriction injury (CCI) rats, and confirmed that exercise can relieve neuropathic pain (NP). They found remarkable differences in the expression of lncRNAs and mRNAs in the CCI rats, and potential biomarkers including Dnah6, Pkd1l2, C3, Adgre1, Plac9, Sgk1, and VGF.  Li, Li et al. also studied three diagnostic biomarkers related to immune cell infiltration, and found that five hub genes involved in the pathogenesis might acted as potential genes for target therapy points of lumbar disc herniation as assessed with the bioinformatics analysis. Perhaps we could replicate or conduct with the similar methodology of the aforementioned research to explore more molecular biomarkers or hub genes to explain the mechanism of pain. In addition, Zhou et al. identified a novel neuron type MAAC (Fxyd7+/Atp1b1+) in dorsal root ganglia of painful diabetic peripheral neuropathy rats with the single-cell RNA sequencing technique, and investigated the transcriptomic characteristics, origin, transition trajectory, regulator and cellular communication. However, this study has not yet thoroughly clarified the role of MAAC in NP.

## Neuromorphological approach

Research on the comorbidity of pain, such as emotional disorders and itch, is still in its infancy. Dai et al. exerted significant differences in transcriptomic characteristics between the medial prefrontal cortex (mPFC) and the anterior cingulate cortex (ACC) of mice after spared nerve injury exhibiting acute pain and mild depression. Interestingly, there was sexual dimorphism at the transcriptional level. Furthermore, this study indicated that restoring oligodendrocytes and myelin in the mPFC or extracellular matrix in the ACC may be key to the treatment of pain-depression comorbidity. Overall, this study further highlights the role of the sexually dimorphic brain in neuropsychiatric disorders by examining differences at the neuromorphological and molecular levels. Accordingly, more research studies may be needed to focus on gender differences and its dependent factors, such as sex hormones and genetics, in the pathogenesis and management of pain or its comorbidities. Li, Bai et al. investigated parvalbumin neurons in the in zona incerta that acts as an endogenous negative modulatory center. Up to date, studies have demonstrated that pain, itch, and depression are intricately entangled at anatomical, circuit and molecular levels (Caccavale et al., 2016; Malfliet et al., 2017; Koch et al., 2018; Belinskaia et al., 2019; Roberts et al., 2019; Mihailescu-Marin et al., 2020; Najafi et al., 2021). Therefore, it is very critical to discover the same core pathogenic factors, such as sensory center, among these three symptoms for the future studies.

## Study of pain sensitivity

Currently, neuroimaging and neural regulation techniques are widely used in pain study. These techniques may include functional magnetic resonance imaging (fMRI), diffusion tensor imaging (DTI), functional near-infrared spectroscopy (fNIRS), repetitive transcranial magnetic stimulation (rTMS), and transcranial direct current stimulation (tDCS). In this present thematic topic, the pain sensitivity assessed with these techniques seems to be an intriguing point. Zou et al. found that the multi-features (regional homogeneity and connectivity metrics) of multimodal MRI (fMRI and DTI) shows more accurately predictive in anticipating the pain sensitivity. In another study of comparing the effects of three rTMS paradigms on different sensory fibers (Aβ, Aδ, and C fibers) assessed with the fNIRS, Li, Zhang et al. revealed that prolonged continuous theta-burst stimulation can modulate sensitivity on Aβ fibers and affect the activation of dorsolateral prefrontal cortex, frontopolar cortex and other brain regions. Li, Lin et al. directly explored how single session of high-definition tDCS (HD-tDCS) over the primary motor cortex (M1) affects experimental pain sensitivity among healthy participants. Results showed that only anodal HD-tDCS significantly increased cold pain threshold. Interestingly, the effectiveness of anodal HD-tDCS in attenuating pain intensity ratings to suprathreshold pain was dependent upon the level of attentional bias to negative information. It highlights those individual differences in pain-related cognition should be taken into consideration in the clinical applications of tDCS for pain relief.

## Disease-specific pain

The neural mechanisms and interventions of specific pain have always been concerned by researchers. Wei, Yang et al. conducted in-depth exploration on migraine without aura (MwoA) and used ML model to study the efficacy of non-steroidal anti-inflammatory drugs (NSAIDs). Their team showed us two high-quality studies on this topic. In Wei, Yang et al., dysfunctional connections within seven networks were observed, including default mode network, executive control network, salience network, sensorimotor network, dorsal attention network, visual network, and auditory network. The support vector machine (SVM) model using the clinical characteristics and functional network connectivity (FNC) abnormalities as features has also proved to be very helpful in predicting efficacy of NSAIDs and improving the decision-making of therapeutic strategy. In Wei, Xu et al., the abnormal functional connectivity (FC) between amygdala and multiple brain regions has been found, which helps to reveal the neural network mechanism of MwoA. At the same time, the multivariable logistic regression (MLR) and SVM models that include disrupted FC patterns from amygdala-based FC analysis and clinical characteristics also show high reliability in verifying the efficacy of NSAIDs, which helps to solve the clinical efficacy problem of MwoA. In other respects, de Souza Moura et al. provided a new study protocol to explore the treatment of head and neck cancer patients under chemoradiation therapy condition with tDCS. The expected results from this proposed study might shed lights on better understanding the neural mechanism of tDCS intervention on the cancer patients.

## Pain study in trend

Our topic also focuses on the latest trends in the field of pain to promote research progress. Li, Shu et al. and Du et al. conducted bibliometric analysis on the field of post stroke pain and diabetic peripheral neuropathic pain, respectively, and provided an analytical method to visualize the trend and frontiers of the above fields. Finally, Yang et al. summarized the research progress of non-invasive brain stimulation (NIBS) in the treatment of different central neuropathic pain (CNP)s and described the effects on alleviating CNPs as well as the underlying mechanisms. It is suggested that the future research should gradually carry out large-scale multi center research to verify the stability and reliability of the analgesic effect induced by NIBS.

To sum up, this paper on the topic “*Neural Networks and Regulatory Mechanisms Associated with Pain*” expands the mechanistic theories, biomarkers and targeted drugs at the neural network and molecular levels in the field of pain.

## Author contributions

All authors listed have made a substantial, direct, and intellectual contribution to the work and approved it for publication.

## Conflict of interest

The authors declare that the research was conducted in the absence of any commercial or financial relationships that could be construed as a potential conflict of interest.

## Publisher's note

All claims expressed in this article are solely those of the authors and do not necessarily represent those of their affiliated organizations, or those of the publisher, the editors and the reviewers. Any product that may be evaluated in this article, or claim that may be made by its manufacturer, is not guaranteed or endorsed by the publisher.

## References

[B1] Belinskaia D. A. Belinskaia M. A. Barygin O. I. Vanchakova N. P. Shestakova N. N. (2019). Psychotropic drugs for the management of chronic pain and itch. Pharmaceuticals 12:99. 10.3390/ph12020099 31238561 PMC6631469

[B2] Caccavale S. Bove D. Bove R. M. (2016). Skin and brain: itch and psychiatric disorders. G. Ital. Dermatol. Venereol. 151, 525–529. Available online at: https://www.minervamedica.it/en/journals/Ital-J-Dermatol-Venereol/article.php?cod=R23Y2016N05A0525 25854671

[B3] Koch S. C. Acton D. Goulding M. (2018). Spinal circuits for touch, pain, and itch. Annu. Rev. Physiol. 80, 189–217. 10.1146/annurev-physiol-022516-034303 28961064 PMC5891508

[B4] Malfliet A. Coppieters I. Van Wilgen P. Kregel J. De Pauw R. Dolphens M.. (2017). Brain changes associated with cognitive and emotional factors in chronic pain: a systematic review. Eur. J. Pain 21, 769–786. 10.1002/ejp.1003 28146315

[B5] Mihailescu-Marin M. M. Mosoiu D. V. Burtea V. Sechel G. Rogozea L. M. Ciurescu D. (2020). Common pathways for pain and Depression-Implications for practice. Am. J. Ther. 27, e468–e476. 10.1097/MJT.0000000000001235 32897982

[B6] Najafi P. Dufor O. Ben S. D. Misery L. Carre J. L. (2021). Itch processing in the brain. J. Eur. Acad. Dermatol. Venereol. 35, 1058–1066. 10.1111/jdv.17029 33145804

[B7] Roberts C. A. Giesbrecht T. Stancak A. Fallon N. Thomas A. Kirkham T. C. (2019). Where is itch represented in the brain, and how does it differ from pain? An activation likelihood estimation meta-analysis of experimentally-induced itch. J. Invest. Dermatol. 139, 2245–2248. 10.1016/j.jid.2019.04.007 31054845

